# Special Issue: “Post-COVID-19 Syndrome”

**DOI:** 10.3390/v16121901

**Published:** 2024-12-10

**Authors:** Rüdiger E. Scharf

**Affiliations:** 1 Institute of Transplantation Diagnostics and Cell Therapy, Division of Hemostasis, Hemotherapy, and Transfusion Medicine, Blood and Hemophilia Comprehensive Care Center, Heinrich Heine University Medical Center, D-40225 Düsseldorf, Germany; ruediger.scharf@medma-uni-heidelberg.de; 2 Harvard Medical School, Program in Cellular and Molecular Medicine, Boston Children’s Hospital, Karp Family Research Laboratories, Boston, MA 02115, USA

On 30 January 2020, the World Health Organization declared COVID-19 a Public Health Emergency of International Concern (PHEIC)—the highest WHO warning level. As of 4 May 2023, the WHO Emergency Committee on the COVID-19 pandemic determined that “COVID-19 is now an established and ongoing health issue that no longer constitutes a PHEIC” [1]. Despite this announcement, several serious concerns remain.

*First*, SARS-CoV-2 is not conquered. The virus continues to circulate and can develop novel, even more dangerous variants at any time.

*Second*, by the end of November 2024, more than 776.1 million cumulative cases and about 7.1 million confirmed deaths have been reported to the WHO [2]. However, preliminary WHO estimates suggest that the total number of global deaths attributable to the COVID-19 pandemic is about 20 million, representing a 3-fold higher mortality rate than officially reported [1,2]. In fact, COVID-19 deaths are a key indicator to track the evolution of the pandemic. However, many countries are still lacking functioning civil registration and vital statistic systems with the capacity to record and provide accurate, complete, and timely data on deaths (and causes of deaths). As evident from a recent WHO assessment of health information systems and their capacity in 133 countries, the percentage of registered deaths ranges from 98% in European countries to only 10% on the African continent, and data from the Russian Federation are not available [3].

*Third*, apart from these limitations and uncertainties, registries operated by other institutions than WHO have terminated the collection of COVID-19 data. For example, as of 10 March 2023, after three years of around-the-clock tracking of COVID-19 data from around the world, Johns Hopkins has discontinued its Coronavirus Resource Center’s operations.

*Fourth*, subjects who do not recover but suffer from a variety of complaints or experience persistent symptoms, post-acute sequelae of SARS-CoV-2, and/or defined secondary illness after acute SARS-CoV-2 infection represent another significant concern and challenge. Currently, the nature and pathology of this post-COVID condition, also termed long COVID or post-COVID syndrome, are incompletely understood. Consequently, its management may also be inappropriate in current clinical practice.

In fact, there are several seminal questions with regard to the impact of SARS-CoV-2 on health and disease, specifically on post-COVID condition, but, as stated by Professor Steven G. Deeks, an internationally recognized expert on the pathogenesis and treatment of HIV at the University of California, San Francisco, “*we do not have a definition, we do not have a biomarker, we do not have an imaging test, we do not have a mechanism, or a treatment [of post-COVID syndrome]*” [4,5]. There are several substantial reasons to assume that post-COVID syndrome has a multifaceted and multifactorial pathology. Some of the contributing mechanisms, relevant determinants, and possible predictors, such as certain demographic factors, comorbidities, viral load, social determinants, and the vaccination status of affected individuals, are displayed in Figure 1.

This Special Issue on “Post-COVID Syndrome” was designed to address some of these items, to review present knowledge, and to add information provided by original work from current clinical studies. As one of the Special Issue’s guest editors, I am very pleased that several outstanding clinician-scientists agreed to submit a state-of-the-art paper and to contribute to the success of the publication project. Together with my colleague Juan-Manuel Anaya, Bogotá, Colombia, I am proud of the high quality of the manuscripts that we received. We therefore express our thanks to the authors who also accepted strict timelines, and most of the authors completed their contribution prior to the deadline. We also acknowledge the substantial work (and time) of the referees whose comments and suggestions made the submitted manuscripts even better. Our thanks also go to the members of the Editorial Office and their professional assistance throughout the project.

Finally, we are pleased about the decision by MDPI to publish this series of papers in book format. This effort appears to be more than justified. Thus, we have proudly observed that the majority of the open access papers is being viewed at a high rate. Until the end of November 2024, five top papers received more than 9900 cumulative hits (range, 4038 to 9966 hits per paper), and all papers of this series are being cited increasingly by other investigators. Thus, we trust that the printed version of the Special Issue will foster the sustainability of this publication project and promote the dissemination of valuable information.

Now, as this series of papers is complete, we are convinced that the readers will take benefit from this Special Issue with regard to scientific information, clinical practice, and, most importantly, present and future patient care. In the following paragraphs, some “appetizers” are provided to the readers by highlighting the key information from each article of this series.

An overview covering various aspects of post-COVID syndrome, including prevalence, sequelae, risk determinants, and psychosocial implications, is presented by the editors Rüdiger E. Scharf and Juan-Manuel Anaya [6]. Despite an incomplete understanding, the authors also review the pathogenesis of post-COVID syndrome in detail and specifically discuss hyperinflammation, immuno-thrombosis, and thrombo-inflammation upon SARS-CoV-2 infection. Moreover, the impact of vaccination on the prevention and treatment of post-COVID syndrome as well as the genetic basis of individual susceptibility to and the severity of COVID-19 are reviewed. Autoimmunity is a frequent pathology associated with post-COVID syndrome, possibly induced by misdirected immune processes in response to SARS-CoV-2. Facing the high prevalence of COVID-19 cases worldwide, the authors therefore assume that autoimmune disorders will increase globally in the near future.

In their narrative review on post-acute COVID disorders, Celina Silvia Stafie et al. focus on conditions that can induce a multisystem inflammatory syndrome (MIS) both in children (MIS-C) and adults (MIS-A) [7]. It is emphasized that post-COVID syndrome should be differentiated from MIS, which is characterized by an acute condition with cardiovascular complications and endocrine or metabolic changes. The authors conclude that post-COVID syndrome and MIS represent two pathologies that are connected by an interdependent relationship.

Michal Chudzik et al. retrospectively assessed clinical features and risk factors of the post-COVID condition, using a national registry that includes both inpatients and post-hospitalization patients [8]. The investigators identified chronic fatigue, persistent cough, and a variety of neuropsychological symptoms as the most common features in patients with post-COVID syndrome. The female gender, severe courses of the acute SARS-CoV-2 infection, dyspnea, chest pain, olfactory dysfunction, and arthralgia were shown to predispose patients to the development of post-COVID syndrome.

Patient registries can also provide valuable information on a huge number of items, e.g., prevalences, hospitalization, outcomes, and direct healthcare costs. This is convincingly demonstrated in the report by Nike Walter et al. [9]. The authors analyzed data on more than 543,000 hospitalized patients with COVID-19. In total, nearly 30,000 (5.5%) of them were diagnosed with post-COVID syndrome (PCS). Among PCS patients, 1330 in-hospital deaths were recorded, corresponding to a mortality rate of 4.5%. Total cumulative healthcare costs were estimated at more than 136 million €.

Proper diagnosis and adequate management of post-acute COVID are challenging, specifically since neither a clear definition of what we are searching for nor evidence-based recommendations (not to mention guidelines) for diagnostic process and treatment of post-COVID syndrome currently exist. Therefore, contributions on these issues were particularly welcome to this special edition. In a single-center study, Sarah C. Goretzki et al. have retrospectively evaluated the prevalence of alternative diagnoses in children and adolescents with suspected PCS [10]. To screen the study population for potential PCS, the investigators used a non-validated checklist that relies on the combination of neurological and respiratory symptoms (“clusters”) to assess the probability of PCS versus a differential diagnosis.

In their narrative review, Michael Z. Yan et al. report on the diagnostic process in post-COVID conditions upon evaluation based on triple assessment, including clinical symptoms, monitoring of biomarkers, and diagnostic imaging findings [11]. Specifically, high-risk features with regard to cardiorespiratory complications, vigilance disorders, or psychiatric alterations are discussed. Based on their experience, the authors suggest that an integrated care system consisting of holistic healthcare pathways, detection of system-specific complications, management of mild symptoms, and tailored rehabilitation services is required in the future.

A similar focus but a different approach is described in the article by Andrea Fabbri et al., who review the spectrum of sequelae after SARS-CoV-2 infection specifically in patients with a severe course of the disease [12]. Most common symptoms include breathing problems, changes in taste and/or smell, fatigue, and neuropsychological alterations. Female patients appear to be affected more frequently. Reliable predictors of onset and persistence of the sequelae do not exist.

Thromboinflammation, a characteristic feature of severe SARS-CoV-2 infection, is caused by dysregulated activation of hemostasis and aberrant immune responses of innate defense mechanisms [6]. A reliable biomarker of exuberant hemostatic activation is the plasma level of D-dimers. A significant increase in D-dimers is frequently recorded in *hospitalized* patients with COVID-19 and may have prognostic relevance. Christa Meisinger et al. now report that nearly 15% of patients among an *outpatient* study population displayed persistently elevated D-dimers even several months after the acute illness [13]. However, this observation was not associated with chronic symptoms or long-term sequelae.

To assess the risk of pulmonary fibrosis following COVID-19, Rosario Fernández-Plata et al. conducted a longitudinal prospective study among a limited number of infected healthcare professionals [14]. The investigators report that initial pneumonia and persistently positive SARS-CoV-2 PCR testing (after 4 weeks of the acute illness) were associated with post-COVID pulmonary fibrosis. Consequently, the authors conclude that long-term monitoring is indicated in this patient group.

Cardiac complications are a common feature of acute and post-acute COVID-19. The manifestations and sequelae include cardiomyopathy, myocardial infarction, arrhythmias, and heart failure. In their top-rated article, Aydin Husenov et al. provide a comprehensive overview of cardiac manifestations during and after SARS-CoV-2 infection with a focus on cardiac arrhythmias, including their prevalence, pathogenesis, diagnosis, and treatment [15]. Specifically, the authors discuss direct and indirect arrhythmogenic effects of inflammatory cytokines. An autoimmune response to myocardial antigens due to molecular mimicry appears to be another possible mechanism for cardiac damage in the post-COVID condition. With regard to SARS-CoV-2-associated cardiac complications, observational studies on individuals without structural heart disease, such as young athletes, are of particular interest. Fortunately, the reported rate of post-COVID cardiac arrhythmias is rather low in this subpopulation.

Akash Srinivasan et al. review several studies on acute cardiac complications and post-acute cardiac sequelae [16]. The authors demonstrate that the severity of acute COVID-19 alone is not a reliable risk factor of heart complications in the post-acute phase. There appears to be an association between female gender and persisting COVID-19 symptoms; however, it remains unclear whether women are more likely to experience cardiac manifestations of post-COVID syndrome.

Neuropathic and cognitive disorders with a broad spectrum of symptoms are also a common feature of the post-COVID condition [6]. Here, Inge Kirchberger et al. report on cognitive dysfunction, assessed by objective testing and self-reporting among patients about nine months after infection with SARS-CoV-2 with a rather mild course [17]. About 22% and 25% of the study participants complained of memory and concentration problems, respectively, and objective testing using the revised Impact of Event Scale (IES-R) revealed that more than 55% of the study population had cognitive disabilities. The authors therefore recommend extended cognitive diagnostics and training for affected individuals.

César Fernández-de-las-Peñas et al. used established questionnaires for self-reporting to assess neuropathic pain, anxiety and depression levels, as well as psychological, cognitive, sensitization-associated symptoms, and quality of life data in patients who had recovered from acute SARS-CoV-2 infection [18]. Remarkably, more than 25% of the study patients indicated neuropathic post-COVID pain symptoms, and anxiety levels were significantly higher in females than in males.

Sensory deficits of the olfactory, gustatory, hearing, and vestibular systems are frequent symptoms of post-COVID syndrome [6]. Sonja Ludwig et al. evaluated the presence and duration of sensory dysfunction among 50 subjects up to six months after acute SARS-CoV-2 infection [19]. Most patients reported taste (84%), smell (78%), balance (48%), and/or hearing (16%) being affected. Objective measurements confirmed these data. For example, olfactory testing revealed that 72% of the study patients suffered from hyposmia and 4% from anosmia. In the majority of patients, the dysfunction was persistent even after up to six months, and females were significantly more affected by sensory impairment than males.

Vaccine-induced immune thrombotic thrombocytopenia (VITT), a rare but life-threatening complication following administration of adenovirus-based vaccines against SARS-CoV-2, is mediated by autoantibodies to platelet factor 4 (PF4)-polyanion complexes, as initially reported by Greinacher et al. [20] and others [21,22]. Victoria Panagiota et al. report on the acute management and outcome of nine patients with VITT in a median follow-up observation of 300 days [23]. All patients survived the acute phase; one patient died from long-term neurological sequelae of cerebral venous sinus thrombosis, and seven of the eight survivors fully recovered. The authors also report that long-term management of this highly prothrombotic condition with direct oral anticoagulants appears to be safe and effective.

Overall, post-COVID syndrome is characterized by a wide range of non-specific symptoms, with fatigue, exertion intolerance, concentration problems, and sensory dysfunction being the most common complaints. A systematic review and comprehensive meta-analysis of 194 studies, including 735,006 participants, estimated that, at a follow-up ranging from ≥28 to 387 days, 45% of COVID-19 survivors had at least one unresolved symptom [24]. While disease courses in the early months post-infection have been explored and described in detail, long-term health consequences for patients suffering from post-COVID syndrome have so far remained unclear. A recent prospective observational study from the Charité, Berlin, documents that post-COVID syndrome can persist beyond 20 months post-infection and encompass the full scope of post-infectious myalgic encephalomyelitis/chronic fatigue syndrome [25]. However, the true prevalence of post-COVID syndrome is likely to be lower (i.e., about 6 to 10%) than previously estimated [26].

The editor trusts that this compilation of reviews and articles on various epidemiological, pathophysiologic, diagnostic, and therapeutic aspects of post-COVID syndrome is useful and stimulating for both clinicians and scientists from different disciplines.

## Figures and Tables

**Figure 1:**
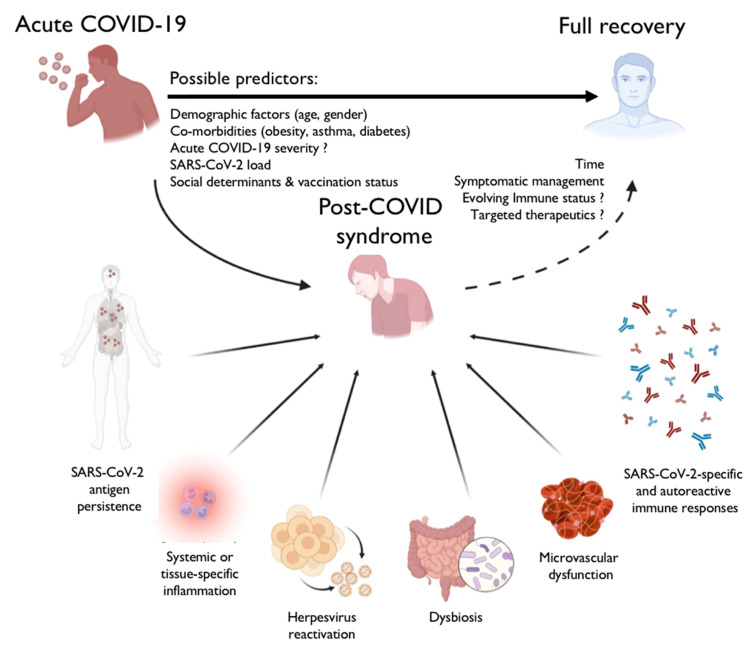
Synopsis of proposed pathophysiologic mechanisms, relevant determinants, and possible predictors of post-COVID syndrome. Of note, among these mechanisms, the persistence of SARS-CoV-2 and/or reactivation of human herpesvirus is discussed. Viral persistence (or reactivation) can trigger autoimmunity (e.g., by molecular mimicry) and cause subsequent pathologies leading to distinct autoimmune disorders (for review: [6]). Treatment of post-COVID syndrome is currently more or less limited to symptomatic modalities. Modification of a scheme taken from Peluso and Deeks [5].
